# Cellulose Nanofibrils Filled Poly(Lactic Acid) Biocomposite Filament for FDM 3D Printing

**DOI:** 10.3390/molecules25102319

**Published:** 2020-05-15

**Authors:** Qianqian Wang, Chencheng Ji, Lushan Sun, Jianzhong Sun, Jun Liu

**Affiliations:** 1 Biofuels Institute, School of the Environment and Safety Engineering, Jiangsu University, Zhenjiang 212013, China; wqq@ujs.edu.cn (Q.W.); cecilji@amecnsh.com (C.J.); junliu115142@ujs.edu.cn (J.L.); 2 Institute of Textiles and Clothing, The Hong Kong Polytechnic University, Hong Kong, China

**Keywords:** melt extrusion, 3D printing, cellulose nanofibrils, biocomposite filaments, physical property

## Abstract

As direct digital manufacturing, 3D printing (3DP) technology provides new development directions and opportunities for the high-value utilization of a wide range of biological materials. Cellulose nanofibrils (CNF) and polylactic acid (PLA) biocomposite filaments for fused deposition modeling (FDM) 3DP were developed in this study. Firstly, CNF was isolated by enzymatic hydrolysis combined with high-pressure homogenization. CNF/PLA filaments were then prepared by melt-extrusion of PLA as the matrix and CNF as the filler. Thermal stability, mechanical performance, and water absorption property of biocomposite filaments and 3D-printed objects were analyzed. Findings showed that CNF increased the thermal stability of the PLA/PEG600/CNF composite. Compared to unfilled PLA FDM filaments, the CNF filled PLA biocomposite filament showed an increase of 33% in tensile strength and 19% in elongation at break, suggesting better compatibility for desktop FDM 3DP. This study provided a new potential for the high-value utilization of CNF in 3DP in consumer product applications.

## 1. Introduction

From aerospace engineering to the maker communities, 3D printing (3DP), as a form of direct digital manufacturing and additive manufacturing, has gained exponential growth in a variety of fields during the last few decades [1,2,3,4,5]. Many have found promising potential in its mass adoption resulting in products such as furniture and architectural design that use various 3DP technologies. Among them, the apparel and textile fields have already been using 3DP in footwear and accessory products that require less flexibility in the 3DP materials applied [6]. Considering the unique advantages of design efficiency and customization in 3DP, some industry brands and professional designers and engineers have been exploring its integration into more wearable textile and apparel products [7,8]. Through unique 3D computer-aided design (CAD) techniques and approaches [9,10], garments developed with 3DP components are now much more versatile and even resilient towards the human body’s movements and fitting. However, few examples are found for the use of a sustainable material base in 3DP-integrated textiles and apparel. Currently, the textile and apparel industry has recognized the negative environmental effects of fiber and fabric production processes, all of which can often involve extensive toxic or biologically modifying reagents [6]. Further, there is a challenge with regards to efficiency and cost in recycling and upcycling postconsumer textile and apparel products [11,12]. Therefore, it is essential that new fabrication technology, such as 3DP, adopts more environmentally sustainable approaches and materials.

Although researchers have begun to explore recycled 3DP materials [13], biocomposite materials used in desktop fused deposition modeling (FDM) 3DP are expected to also become highly impactful in the development of bio-based materials for consumer products [2]. The biomaterials are often derived from wood or agricultural waste. Polylactic acid (PLA), commonly made using corn starch and sugar cane has been one of the FDM thermoplastic options. However, as the 3DP technology rapidly advances to integrate into fields like textiles and apparel, conventional virgin PLA filament can no longer meet the need for diverse products. To improve the physicochemical properties of PLA for FDM 3DP, some researchers have begun to introduce biomass raw materials into the PLA matrix. The addition of microcrystalline cellulose (MCC) as a reinforcing material to the PLA matrix has improved the crystallinity and the strength of the composite material as compared to that of virgin PLA [14,15].

Further, nanocellulose has also been studied as a key additive to strengthen PLA. It is produced from natural cellulose. Its low density, ultra-fine structure, and high strength properties are attractive in the field of reinforcing materials [16]. In a small proportion, nanocellulose can improve the PLA matrix in crystallinity, mechanical properties, degradability, etc. [17]. Scholars have further recognized that 3D printing with nanomaterials could be used in leveraging greater control in fundamental material properties, providing multifunctionality and custom geometry to product needs [18]. Wang et al. [19] prepared micro-nanocellulose using a colloid mill and combined it into the PLA matrix at a relatively high proportion. The developed composite filament had a higher tensile strength.

Additionally, the preparation of nanocellulose is critical in composite development and mainly includes chemical, mechanical, and biological methods [20,21,22]. Nanocellulose prepared by a concentrated sulfuric acid hydrolysis is poor in thermal stability due to the introduction of sulfate half ester groups [23]. Nanocellulose can also be prepared by high-pressure homogenization with long fibers as raw materials. Mechanical homogenization requires high energy consumption. Moreover, the high-pressure homogenizer was occasionally blocked by the long fiber, which led to the failure to discharge. This limitation still exists in the PLA/cellulose nanofibrils (CNF) composite technique when applied to 3D printing processing. Particularly, the uniform dispersion of nanocellulose into the PLA matrix is a real challenge that will significantly affect the printability of the fabricated composite filament [24] and the physical properties of 3D-printed architectures. In addition, PEG, as a plasticizer, commonly used in the melting process for polymers, is another important factor involved in biocomposite fabrication.

**Figure 1:**
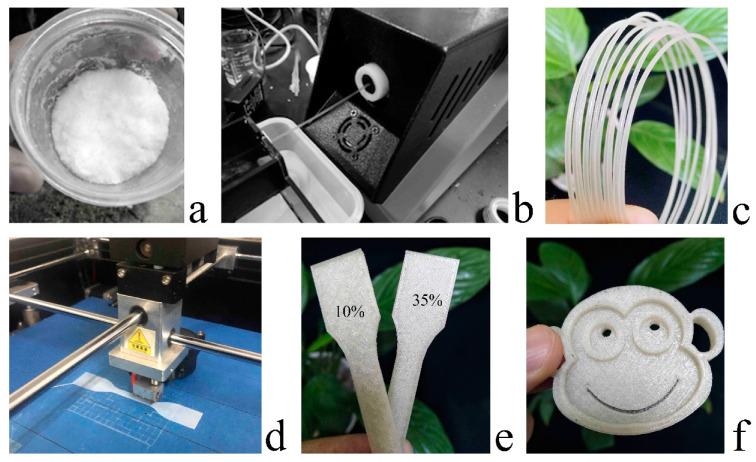
Cellulose nanofibrils (CNF) powder: (**a**) composite filament extruded on Wellzoom desktop extruder; (**b**) composite filament (1.75 mm diameter); (**c**) specimen in printing on M3036 FDM desktop 3D printer; (**d**) 3D-printed specimen with 10% and 35% infill; (**e**) 3D-printed specimen monkey face with 40 mm/s printing speed and 100% infill (**f**).

In this study, the enzymatic hydrolysis of MCC was used to a pretreatment step for high-pressure homogenization. The obtained CNF was freeze-dried. The PLA/CNF composite filaments for 3DP were prepared by melt-extrusion as shown in Figure 1. The properties of the composite filament were characterized and its printability with an FDM 3D printer was verified. The kinetic thermal behavior of the CNF filled PLA filaments were systematically investigated in our previous study [25]. The purpose of this study is to develop CNF filled PLA biocomposite filaments for 3D FDM printing with CNF as the filler, polyethylene glycol 600 (PEG600) as the smoothing agent, and virgin PLA granules as the host matrix. The isolated CNF was tested through morphological studies. The PLA/CNF composite specimens were evaluated in thermal, mechanical, and water absorption tests. The best-performing PLA/CNF composite specimen was extruded into 3DP filament and then used in 3D printing for prototype objects.

## 2. Results

### 2.1. CNF Morphological Observation

**Figure 2:**
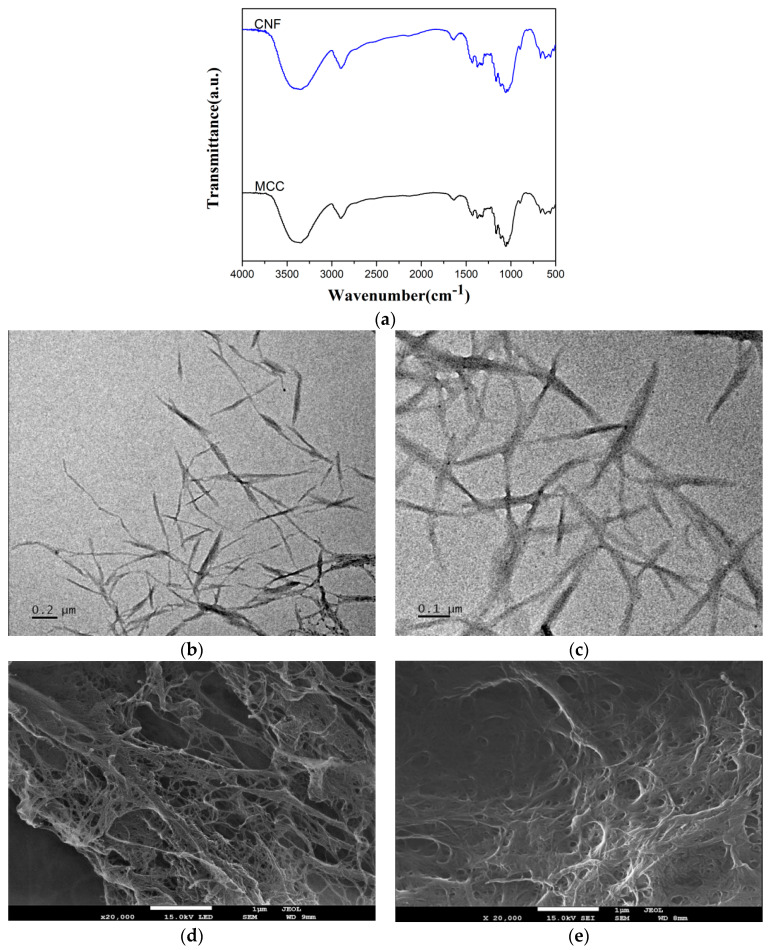
FTIR Spectrum of microcrystalline cellulose (MCC) and CNF. (**a**) TEM images of enzymatic hydrolysis of cellulose with high-pressure homogenization under different scales. (**b**) and (**c**) after 10 passes through the homogenizer. FE-SEM micrographs of CNF at a magnification of 20,000 (**d**) after freeze-drying and (**e**) after mechanical dispersion (following freeze drying).

As can be seen from the TEM images (Figure 2c), the CNF was successfully isolated with 10 passes using enzymatic hydrolyzed MCC. The length of the CNF was in the range of a few hundred nanometers and 1 micron and the diameter was less than 50 nm. This indicated that CNF can be obtained via the combined enzymatic hydrolysis and high-pressure homogenization techniques. The CNF has a large number of hydroxyl groups and a high surface area, thus, a great tendency to form networks during freeze-drying, as shown in Figure 2d. The mechanically milled CNF exhibits a similar network structure (Figure 2e).

The CNF prepared by enzymatic hydrolysis and high-pressure homogenization showed good homogeneity in length and diameter as determined by the statistical analysis of the TEM images. A large CNF fibrous network structure can be seen in the SEM and TEM images. Dried CNF forms complex three-dimensional network structures. The high-pressure homogenization process destroys part of the crystalline structure, resulting in a decrease in the crystallinity of the developed CNF [20]. FTIR spectra (Figure 2a) show typical IR bands for the cellulose structure. The peak at 3450 cm^−1^ was attributed to the O-H group stretching vibration. The peak at 2900 cm^−1^ was derived from the C-H stretching, while the peak at 1060 cm^−1^ was attributed to the vibration of C-O-C in the glucose ring [26].

### 2.2. PLA/CNF Thermal Stability Analysis

In our study, PEG600 is not only working as a plasticizer but also as an adhesive in the filament preparation procedure. The CNF and PLA particles with PEG600 can be homogeneously mixed at a temperature above the PEG600 melting point (17–22 °C). The CNF/PEG600 mixture stuck to the PLA particles when the temperature dropped below the melting point of the PEG600. Without PEG600, the dispersion of CNF in the PLA particles is a real challenge for the single screw extruder. High-quality filaments cannot be prepared without the addition of the PEG600 using the single screw extruder, and only the filaments with high quality are suitable for further analysis.

**Figure 3:**
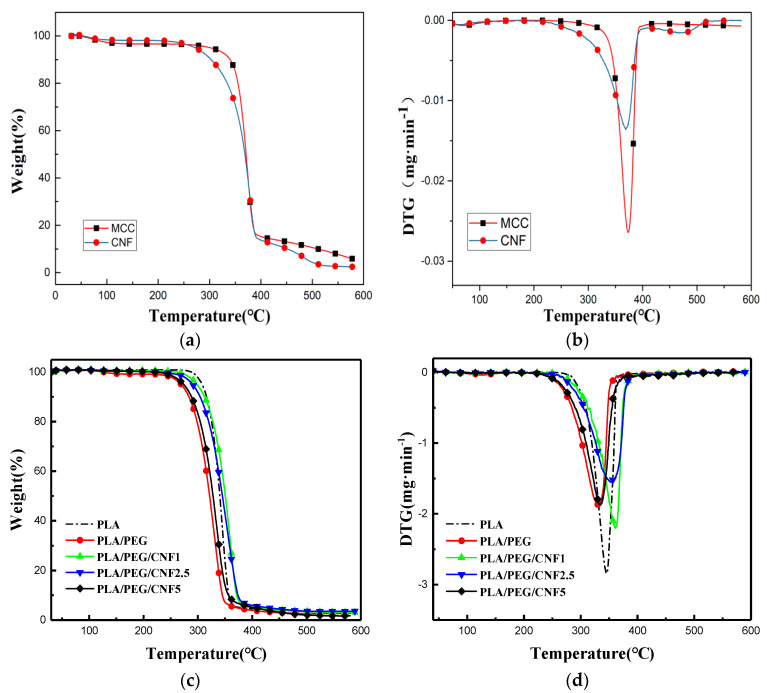
TGA analysis of MCC and CNF: (**a**) TG curves, (**b**) DTG curves; TGA analysis of virgin polylactic acid (PLA) and composites with different wt.% CNF: (**c**) TG curves and (**d**) DTG curves.

In Table 1 the weight loss temperature at 5% wt. of mass loss (T5%) was used as the initial decomposition temperature because we decided that is more reliable than T_onset_. In the TG and DTG curves of the MCC and CNF the peak at 100 °C due to the adsorbed water can be observed (Figure 3a). The values of T_5%_, T_10%_, T_50%_, and T_vmax_ for the CNF are lower than that of the MCC, suggesting that the thermal stability of the CNF is lower than that of the MCC (Table 1).

Compared to composites with and without PEG600 (Figure 3c–d), the thermal stability of the PLA with PEG600 is lower than the virgin PLA. This suggests that the small molecule PEG600 can alter the thermal behavior of the PLA [27], making the intermolecular heat transfer faster and accelerating the break of the molecular chain. Table 1 shows that the virgin PEG600 composite began to decompose at 267 °C, which contrasts with the PLA/PEG600/CNF composite’s initial decomposition temperature that is higher, up to 298 °C at 1 wt.% CNF. Also, the composite’s T_5%_ appears to improve when the CNF is at 1 wt.% that decreases to 273 °C than when the CNF is at 5 wt.%, which is still higher than the virgin PLA and PEG600 composite. The thermal stability of the composite shows the tendency of rising at a low CNF addition and declining as the CNF wt.% increases that suggests the CNF proportion increase results in the gradual change of the composite’s thermal degradation behavior.

The DTG curves (Figure 3d) show that the PLA/PEG600/CNF composite’s maximum rate of weight loss starts at 361 °C and tends to decrease as the CNF wt.% increases. The best thermal stability was found for composites with 1 wt.% of CNF addition.

### 2.3. PLA/PEG600/CNF Composites Mechanical Performance Analysis

**Figure 4:**
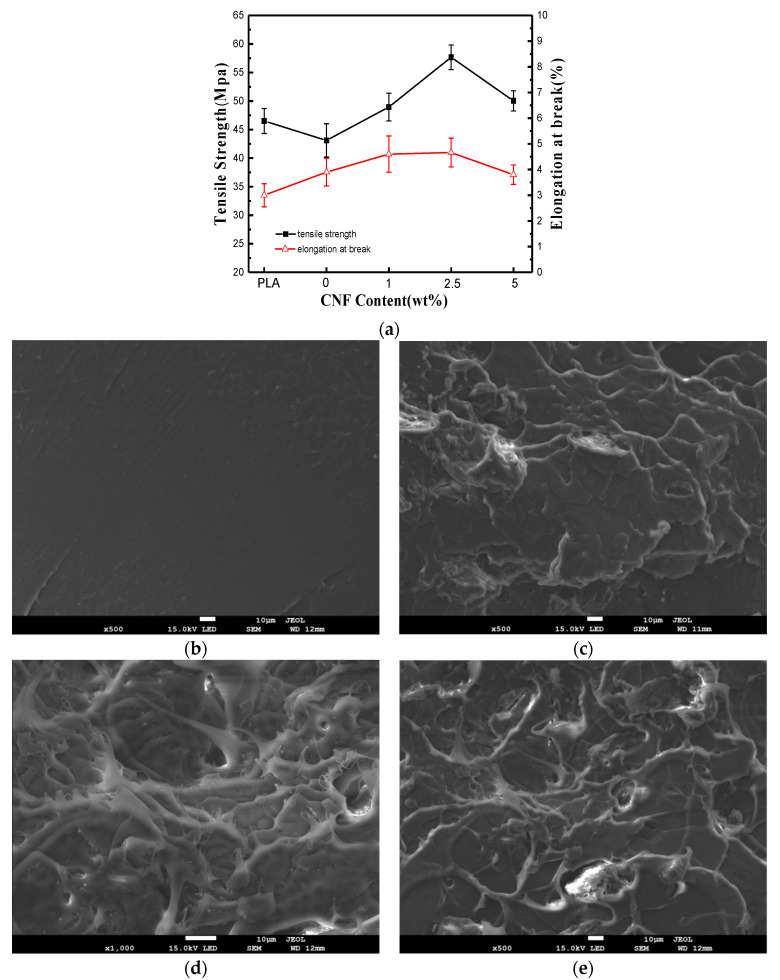
(**a**) Tensile properties of PLA/PEG/CNF composites with different CNF loading; (**b**) FE-SEM micrographs of cross-section of virgin PLA; (**c**) PLA/PEG/CNF 1 wt.%; (**d**) PLA/PEG/ CNF 2.5 wt.%; (**e**) PLA/PEG/CNF 5 wt.%. Scale bar = 10 μm.

Figure 4a shows the tensile strength of the PLA/PEG600/CNF filaments. The addition of PEG600 increased the elongation at break and reduced the tensile strength of the PLA filament. This is due to the plasticizing effect of PEG600. The mechanical properties of the composite material first increased and then decreased with the increase of the CNF content. The maximum mechanical stability was observed at 2.5 wt.% loading. The CNF can be well-dispersed in the PLA matrix thanks to the compatibilizing effect of the PEG600, and the hydrogen bonds could be formed among CNF, PLA, and PEG600, leading to the increase in the tensile strength and elongation at break. When the CNF content is low, the cross-linked structure of the CNF is loose in the composite system, which has little effect on the mechanical properties. When 5 wt.% of CNF is added, the CNF can be partially agglomerated in the PLA matrix, and this becomes a defect point in the composite material. The specimen’s cross section was analyzed by FE-SEM (Figure 4b–e). Compared to the high, flat surface of the virgin PLA, the cross-section of the various CNF composites appeared increasingly uneven with fibrous structures.

### 2.4. PLA/PEG600/CNF Composites Water Absorption

**Figure 5:**
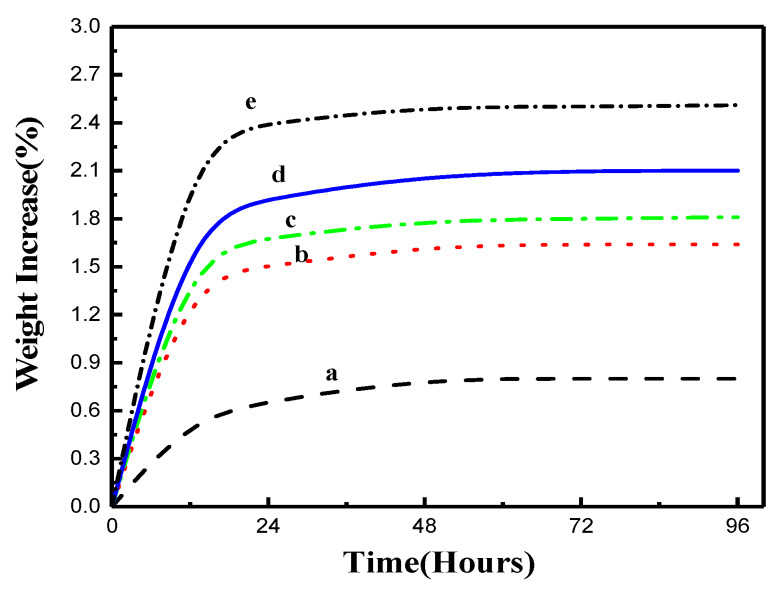
(**a**) Water absorption rate of virgin PLA; (**b**) PLA/PEG; (**c**) PLA/PEG/CNF 1 wt.%; (**d**) PLA/PEG/CNF 2.5 wt.%; (**e**) PLA/PEG/CNF 5 wt.%.

Although water absorption may harm the structural stability of the specimen, it may also have a positive effect on the biodegradability. Since CNF is hydrophilic, it is vital to determine the water absorption rate. In water absorption results, the specimens showed a slight weight increase during the 96 h of the experiment (Figure 5). Overall, compared to the virgin PLA, the composites with CNF at various wt.% showed a similar trend, and absorption of water was the fastest in the first 24 h and stabilized afterward around hour 48 of the experiment. The higher the wt.% of CNF, the higher the water absorption rate of the specimen. Furthermore, the virgin PLA/PEG600 composite reflects a drastic increase in water absorption rate as compared to neat PLA. The water absorption rate increased gradually with the increase of CNF content because the cellulose surface contains a large number of hydrophilic hydroxyl groups. The terminal hydroxyl groups in PEG600 may also affect water absorption rate. Because PEG600 can alter the formation of crystallites of PLA, it allowed more water molecules to bond with hydroxyl groups in the composites’ structure, thus increasing the water absorption rate. PLA degradation is water-based. Thus, the improvement in the water absorption rate may enhance the PLA degradation rate.

## 3. Discussion

### 3.1. Filament Extrusion and 3D Printing

The PLA/PEG/CNF 2.5 wt.% filament was tested in 3D-printed prototypes (Figure 1b,c). Overall, the resulting filaments revealed a smooth surface with improved properties compared to the conventional PLA filament. It suggests the compatibility of this kind of material in desktop FDM 3D printing. In evaluating the surface quality of the composite filament, the developed prototypes (Figure 1b,c) are compared in a simple strip design with 10% and 35% fill rates. The outcome with the higher fill rate (35%) showed higher compactness than the sparse texture in the lower fill rate (10%) prototype, which can be suitable for application needs. In the more complex structure (Figure 1f), the outcome surface remained smooth, and no clogging occurred in the printer nozzle during the printing process.

In terms of the tensile strength and elongation at break for the final filament, the results show that the maximum tensile strength and elongation at break of the PLA/PEG600/CNF composite filaments range from 19.5% to 33.8%, which is higher compared to the pure PLA filament [28]. Two aspects are vital in developing the CNF filled PLA filaments: (1) PEG600 served as a plasticizer in improving the composite’s compatibility. In the post-enzymatic reaction, the higher-pressure homogenized CNF leads to a larger surface area of CNF. Therefore, mixing of the PEG600 with the CNF allowed the additives to blend with the PLA granules more efficiently. It is important to note that PEG600 also provides lubrication in the final filament extrusion. (2) When the CNF wt.% is low (2.5 wt.%), the composites resulted in a looser crosslinked structure that provided minimal impact on the composite’s final tensile strength. On the contrary, when the CNF is high (5 wt.%), the CNF is not able to evenly disperse in the PLA matrix, which leads to agglomeration and weakness of tensile strength. Compared to the pure or conventional PLA, the final PLA/PEG600/CNF composite filaments show the maximum tensile strength and elongation at break of 33.8% and 19.5%, respectively.

### 3.2. Preparation CNF and PLA/PEG600/CNF Composites

To develop high-quality PLA-based composites, several factors are critical.

1) The 2-stage preparation procedure for CNF is prerequisite for the later effective composite mixing. The combined use of enzymatic hydrolysis and high-pressure homogenization enabled efficient CNF modification. Further, previous research has resulted in visible granules in the specimens with high CNF concentration. This may become one of the major limiting factors in the PLA composite’s development [29]. Therefore, the mechanical pretreatment for freeze-dried composite is critical to increase the material dispersion and reduce agglomeration in the PLA composite.

2) The unique use of PEG600 is very important in achieving high performance in the final filament extrusion and 3D printing. The CNF developed already shows higher water absorption and thermal stability. After adding PEG600, these qualities were further enhanced.

3) The CNF used in composite development is often customized but limited in the preparation techniques or recipes in former studies. Previous research [30] employed fine wood powders to mix into the PLA that resulted in a rough and uneven texture. Here, the mixing of the PEG600 is much simpler and cheaper, and largely reduces the need for industrial processing to achieve an even composite mixture. In addition, the properties of the CNF produced by different techniques vary greatly, so it is critical to ensure the stability and quality of the CNF for 3D-printing-related applications.

### 3.3. Textile and Apparel Product Applications

In the textiles and apparel fields, ready-to-wear products and experimental explorations using FDM 3DP are currently limited to thermoplastic materials that are not biodegradable. The PLA/PEG600/CNF biocomposite filament developed in this study indicates great potential for more renewable material in future 3DP-integrated wearables in several ways: (1) Based on the mechanical performance measures, the increased tensile strength and elongation at break provides a foundation for potential 3D-printed structures that can be used in textiles and apparel [7,8]. Many of the currently available filament options for FDM printing have limitations in the material’s overall resilience and durability for comfort considerations. For complex or detailed textile or apparel 3DP structures, it is also important to test the mechanical responses in filament form and outcomes from different printing directions [30]. (2) The hydrophilic nature of the CNF helps to enhance the quality of the developed biocomposite and, more importantly, allows the later coloring or dying post-processing to be more accessible for varying the 3D-printed parts in textile and apparel products.

At a larger scale, (4) the PLA/PEG600/CNF biocomposite filament has the potential of providing a naturally derived material for consumer products and allows higher efficiency in post-consumer recycling and upcycling. (5) It also promotes compatibility in combining with other natural fiber-based traditional fabrics in other applications and design possibilities. However, a limitation still exists in achieving the market-ready quality filament for wearable products. In previous filament explorations, scholars have pointed out the limitations in processing, cost, consistency, volume reliability, and high lead time in nanocomposite production [18]. Further investigation must be conducted to perfect the techniques for higher production yield, as well as its economic performance for various applications.

## 4. Material and Methods

CNF was produced from microcrystalline cellulose (MCC) from the Qu Fu Tian Li Medical Supplement Corporation (Qufu, China). Cellic CTec2 (cellulase cocktail, enzyme blend) was obtained from Novozymes (Suzhou, China), while PEG600 and virgin PLA (4032D) were purchased from Sinopharm Chemical Reagent Limited Company (Shanhai, China) and Natureworks (Minnetonka, MN, USA), respectively. The density of PLA was 1.24 g.cm^−3^ and it had a melting point in range of 155 to 170 °C [31].

### 4.1. CNF Isolation

A 2-stage process was utilized in the CNF preparation. First, the MCC was mixed with acetic acid-sodium acetate buffer solution (pH = 4.6) in 10 wt.% consistency, and 0.5 mL cellic CTec2 (cellulase cocktail, 128 FPU/mL) per gram MCC was added. The enzymatic hydrolysis was kept in a 50 °C water bath for 2 h and inactivated in an 80 °C water bath for 30 min. The resulting cellulose suspension was vacuum filtrated and washed with deionized water. The filtered cellulose suspension was diluted into 2% solid content and homogenized 10 times at 80 Mpa (AH-BASIC, ATS Engineering Co. Ltd., Jiangsu, China). The homogenized cellulose suspension was finally pre-frozen, freeze-dried, and milled to pass through a 300 mesh (Herb Grinder 2, Yongkang Boou hardware products Co. Ltd., Zhejiang, China). The morphology of the resulting CNF was tested with a Tecnai F30 TEM and a Hitachi 7800F FE-SEM. The FTIR spectra of the isolated CNF were determined with a Fourier-transform infrared instrument (Nexus 470, Nicolet Instruments, Offenbach, Germany) in transmission mode. Freeze-dried CNF samples were ground and pelletized using KBr and the spectra were recorded in the range from 400 to 4000 cm^−1^.

### 4.2. PLA/CNF Composite Development and Testing

#### 4.2.1. Preparing the PLA/CNF Composite

The lab for filament extrusion is not air conditioned. The temperature of the lab was around 0 °C in winter. PEG600, which is in a solid state at 0 °C, was heated to liquid form and added into the PLA granules at 4% loading to provide a more even and well-blended consistency. CNF powder was then added at three different wt.% (1, 2.5, and 5) to the final granule mixture for refrigeration (Figure 1a). The PLA/PEG/CNF mixture was melt-extruded using a Wellzoom desktop single screw extruder specially designed for manufacturing 3D printing filaments by Shenzhen Si Da Technology Limited (175 °C at mixing region and 170 °C at extrusion region). The speed of the single screw extruder was set to 35 rpm. A 1.8 mm round hole is arranged at the front of the cooling water tank to control the diameter of the filament and the speed of the extractor was tuned manually according to the speed of the extruder. The resulting filament was maintained with a diameter of 1.75 ± 0.05 mm and was air-cooled and water-cooled. Total of five specimens (virgin PLA, PLA/PEG, PLA/PEG/CNF 1 wt.%, PLA/PEG/CNF 2.5 wt.%, PLA/PEG/CNF 5 wt.%) were used in the following tests.

#### 4.2.2. Thermal Stability

The specimen (~5 mg) was analyzed in an alumina crucible by thermal gravimetric analysis (TGA) for its thermal stability using the temperature range of 30–600 °C at a heating rate of 10 °C/min under nitrogen atmosphere, and the gas flow rate of 20 mL/min.

#### 4.2.3. Mechanical Performance

Tensile strength was measured with a JF-9003 tensile tester by Dongguan Jianfeng Instrument Limited with a gauge of 50 mm, and a speed of 5 mm/min. Filaments with various formulations were tested at least 5 times each and the average value was reported.

#### 4.2.4. Water Absorption

The specimens were first oven-dried at 60 °C for 48 h and weighted, and then submerged in PBS buffer (Phosphate-buffered saline) with pH 7.4 for 24 h at room temperature (20 °C). The specimen was then surface-cleaned with absorbent paper and weighted with an analytical balance (Sartorius TE214S with an accuracy of 0.1 mg, Goettingen, Germany). The water absorption rate was calculated using the following equation [32]:

water absorption rate = [(*M*_2_ − *M*_1_)/*M*_1_] × 100% (1)

Here, the *M*_1_ represents pre-absorbent mass, and *M*_2_ is the post-absorbent mass. The results were reported as an average of two measurements.

#### 4.2.5. 3D Printing and Evaluation

The fabricated filaments were used in printing prototypes for evaluation. The M3036 FDM desktop 3D printer by Shenzhen Technology Limited Co. and Cura slicing software was used to process the CAD models. As for the 3D printing processing, a 0.4 mm nozzle was used. The extruder temperature and printing speed were set as 210 °C and 40 mm/s, respectively. A simple model sample was compared at different fill rates (10%, 35%), and a complex model sample was printed for printability evaluation.

## 5. Conclusions

In this paper, PLA/PEG600/CNF biocomposite filaments were developed and tested for desktop FDM 3D printing. The CNF was prepared using a 2-stage technique involving enzymatic hydrolysis with high-pressure homogenization. PEG600 was added to the composite to improve material performance. The final PLA/PEG600/CNF composite material was extruded to 3DP filament and printed into prototypes. With a CNF content of 2.5 wt.%, the test results showed that the composite filament can meet the requirements of desktop FDM 3D printing. The water absorption rates of CNF composites may indicate good structural stability in a humid environment. The study further pinpoints some valuable properties for applications in the highly prospected textiles and apparel industry. The potential advantage in using the biodegradable 3DP composite in relevant products could lead to higher energy-efficient processing in product recycling and upcycling. Traditional fabric customization by 3D printing may be possible with less labor-intensive processing and could be explored in a future study. Further studies should evaluate the functional and comfort properties and potential of the 3D-printed parts, particularly in different textile and apparel product integrations. Additionally, the effects of infill structures in the 3D-printed parts should be considered for different functional applications. Future studies can also expand to the fields of architecture, interiors, and packaging applications. More efforts in structural design, 3D printing process optimization, and post-processing are needed to increase the mechanical performance in nanosized natural fiber-reinforced PLA filament and 3D-printed products.

## Figures and Tables

**Table 1:** Weight loss temperature of MCC, CNF, PLA, PLA/PEG, and PLA/PEG/CNF composites.

Specimen *	T_onset_(°C)	T_5%_(°C)	T_10%_(°C)	T_50%_(°C)	T_vmax_(°C)
MCC	353	301	340	371	372
CNF	321	257	292	362	369
PLA	311	308	316	340	346
PLA/PEG	295	267	284	321	330
PLA/PEG/CNF 1 wt.%	320	298	312	350	361
PLA/PEG/CNF 2.5 wt.%	311	290	304	344	352
PLA/PEG/CNF 5 wt.%	302	273	288	328	335

*: PLA/PEG/CNF 1 wt.%, PLA/PEG/CNF 2.5 wt.%, PLA/PEG/CNF 5 wt.% means CNF loading were 1, 2.5 and 5 wt.%, respectively.
